# Distal end side-to-side anastomoses of sequential vein graft to small target coronary arteries improve intraoperative graft flow

**DOI:** 10.1186/1471-2261-14-65

**Published:** 2014-05-09

**Authors:** Haitao Li, Baodong Xie, Chengxiong Gu, Mingxin Gao, Fan Zhang, Jiayang Wang, Longsheng Dai, Yang Yu

**Affiliations:** 1 Department of Cardiac Surgery, Beijing An Zhen Hospital, Capital Medical University, Beijing 100029, China; 2 Department of cardiovascular surgery, The second affiliated hospital of Harbin Medical University, 246 Xuefu Road, Harbin, Heilongjiang 150086, China

**Keywords:** Coronary artery bypass grafts, venous graft, off-pump surgery

## Abstract

**Background:**

End-to-side anastomoses to connect the distal end of the great saphenous vein (GSV) to small target coronary arteries are commonly performed in sequential coronary artery bypass grafting (CABG). However, the oversize diameter ratio between the GSV and small target vessels at end-to-side anastomoses might induce adverse hemodynamic condition. The purpose of this study was to describe a distal end side-to-side anastomosis technique and retrospectively compare the effect of distal end side-to-side versus end-to-side anastomosis on graft flow characteristics.

**Methods:**

We performed side-to-side anastomoses to connect the distal end of the GSV to small target vessels on 30 patients undergoing off-pump sequential CABG in our hospital between October 2012 and July 2013. Among the 30 patients, end-to-side anastomoses at the distal end of the GSV were initially performed on 14 patients; however, due to poor graft flow, those anastomoses were revised into side-to-side anastomoses. We retrospectively compared the intraoperative graft flow characteristics of the end-to-side versus side-to-side anastomoses in the 14 patients. The patient outcomes were also evaluated.

**Results:**

We found that the side-to-side anastomosis reconstruction improved intraoperative flow and reduced pulsatility index in all the 14 patients significantly. The 16 patients who had the distal end side-to-side anastomoses performed directly also exhibited satisfactory intraoperative graft flow. Three-month postoperative outcomes for all the patients were satisfactory.

**Conclusions:**

Side-to-side anastomosis at the distal end of sequential vein grafts might be a promising strategy to connect small target coronary arteries to the GSV.

## Background

An important technical challenge for a cardiac surgeon is to perform high quality anastomoses to small target vessels in coronary artery bypass grafting (CABG). In many Chinese patients, the target coronary arteries available for grafting are small due to either lesion accumulation or intrinsic vessel characteristics. In sequential CABG, an end-to-side anastomosis is usually performed to connect the distal end of the graft and a target coronary artery. However, the diameters of grafts and target vessels are frequently unmatched. Spivack et al. reported that the mean diameter of the great saphenous vein (GSV), which is commonly used for sequential CABG, is between 2.3 mm to 4.4 mm [1]; while the average diameter of small target coronary arteries of Chinese patients is usually 1 mm. Graft-host diameter ratio has been shown to influence distal anastomotic hemodynamics and consequently graft patency [2-4]. Although large graft-host diameter ratio has been shown to have better hemodynamic performance than smaller ones under end-to-side anastomoses [5], the oversize diameter ratio between the GSV and small target coronary arteries might induce adverse hemodynamics condition and compromise graft patency. Side-to-side anastomoses, which have been shown to produce favorable hemodynamic flow patterns as compared to end-to-side anastomoses [6-12], might be an option for connecting the distal end of the GSV to small target vessels. The purpose of this study was to introduce a technique of distal end side-to-side anastomosis and compare the effect of side-to-side versus end-to-side anastomosis on intraoperative graft flow characteristics in sequential CABG.

## Methods

### Patients

The study was approved by the Institutional Ethics Committee of Beijing An Zhen Hospital. All patients signed the informed consent. Thirty Chinese patients, who underwent sequential off-pump CABG surgery in the Department of Cardiac Surgery of Beijing An Zhen Hospital between October 2012 and July 2013, participated in this study. All operations were conducted by the same surgeon, Dr. Chengxiong Gu. The patient characteristics were displayed in Table 1.

**Table 1:** Patient characteristic

**Number of patient**	30	
**Men**	19	63.3%
**Women**	11	36.7%
**Age**	68 ± 3.6 (63–73)	
**Number of diabetes**	16	53.3%
**Number of hypertension**	20	66.7%
**New York Heart Association Function Classification**		
**I**	0	0
**II**	8	26.7%
**III**	13	43.3%
**IV**	9	30%

### Surgical technique

All operations were performed through median sternotomy. The GSV was used for sequential grafting in operations. The estimated diameter of the target coronary arteries to be connected to the distal end of the GSV, either the posterior descending artery (PDA) or the posterior left ventricular branch (PLV), was approximately 1 mm in all the patients. The proximal anastomoses were first completed using a partial occlusion clamp and polypropylene 6–0 sutures. Side-to-side anastomoses were performed with a diamond configuration for the middle anastomoses in the sequential grafts. Initially, in 14 patients the distal end anastomoses were revised using side-to-side anastomosis technique after end-to-side anastomoses had shown unfavorable intraoperative graft flow characteristics. Based on the significant improvement of graft flow after the side-to-side anastomotic reconstruction in the 14 patients, an additional 16 patients with small target coronary arteries had distal end side-to-side anastomoses performed.

**Figure 1:**
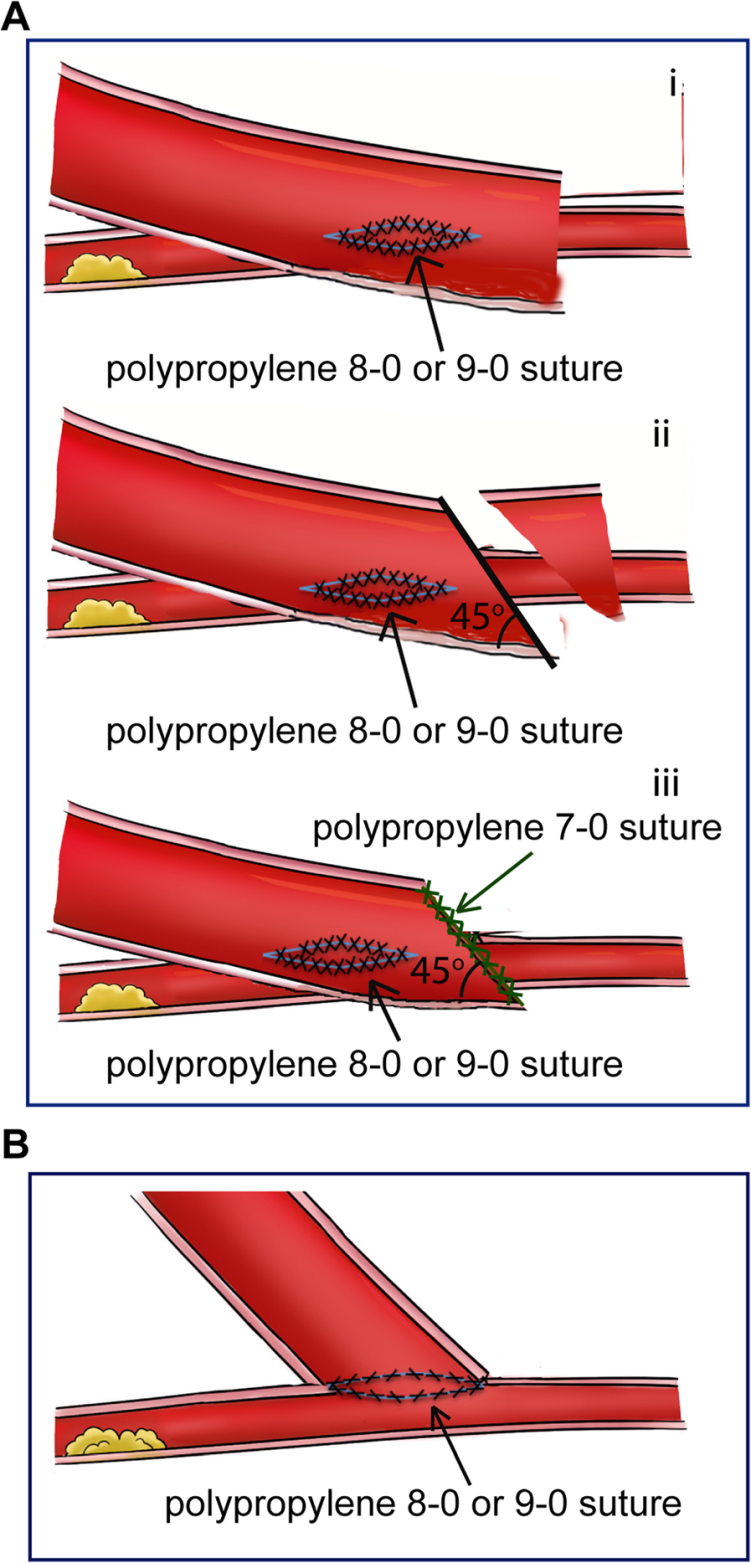
**Schematic diagram of distal end side-to-side anastomoses and end-to-side anastomoses. A**. Schematic diagram of distal end side-to-side anastomoses. i. The GSV was side-to-side anastomosed to the target coronary artery by Prolene 8–0 or 9–0 sutures. ii. The extra GSV segment was subsequently cut starting from the toe of the anastomosis at 45-degree angle toward the anastomosis. iii. The distal end of GSV was sealed with Prolene 7–0. **B**. Schematic diagram of end-to-side anastomoses.

Distal end side-to-side anastomoses of the GSV to target vessels were performed in the following steps. The length of the GSV was kept about one centimeter longer than required. The distal end of the GSV was first left open and side-to-side anastomosed to the target vessel. Target vessels were stabilized by suction type myocardial stabilizer (Recovery Co., Ltd., Beijing, China). The incisions on the GSV and the target coronary artery were parallel to the long axis and approximately 3 mm long. The side-to-side anastomosis was completed using polypropylene 8–0 suture. In the cases that target vessels have thin wall, 9–0 suture was used. According to our experience, the coronary arteries of Chinese patients are small and sometime have thin walls. Thus, small needles and sutures are frequently used in our hospital. The surgeon wore a 2x magnification surgical loupe to perform the anastomosis. A double ended suture needle was used. The suturing technique began at the heel position. The suture was made from outside to inside of the graft. Consequently the next suture was made at the corresponding point of target vessel from inside to outside of target vessel. The extra GSV segment beyond the anastomosis was subsequently cut starting from the toe of the anastomosis at 45-degree angle toward the anastomosis (Figure 1A). The distal end of GSV was sealed with polypropylene7-0 suture (Figure 1A). To compare side-to-side with end-to-side anastomosis configuration, a schematic diagram of standard end-to-side anastomoses was illustrated in Figure 1B.

### Transit time flow measurement

Flow parameters including graft blood flow (BF) and pulsatility index (PI) were measured intra-operatively using a transit time flow meter (VeriQ, Medistim Inc, Oslo, Norway) after all anastomoses were completed. The measurement was performed twice for each graft. One measurement was performed by placing the flow probe at 2 cm distal to the aortic anastomosis, and the second measurement was performed by placing the flow probe at 2 cm proximal to the distal end anastomosis of a graft. During the operation, coronary dilators were discontinued. Intraoperative graft flow and PI were measured when blood pressure became stable.

### MB isoenzyme of serum creatine kinase (CK-MB) measurement

Serial samples of blood, approximately 5 ml each, were drawn on arrival to the surgical ICU (“post-op”), at 6 h, 24 h, and 48 h after surgery. CK-MB level was analyzed using a commercially available kit (Roche Diagnostics, Indianapolis, Indiana). The conventional threshold of this assay for the diagnosis of myocardial necrosis at our hospital is 6.9 ng/ml. The assay was performed on an Elecsys 1010 platform (Roche Diagnostics, Indianapolis, Indiana).

### Follow-up examination

All the patients came back to follow-up visit three months after the operation. During the follow-up, the cardiac function of the patients was evaluated by electrocardiography and echocardiography.

### Statistical analysis

Data were presented as mean ± standard deviation and analyzed using the statistical analyzing software SPSS (SPSS Inc, Chicago, IL). Pairwise comparison was performed by two-tail Student’s *t-*test. P < 0.05 was considered statistically significantly different.

## Results

The first 14 patients operated with the new technique had revisions due to poor performance of an end-to-side anastomosis at the distal end of the GSV. The intraoperative graft flow parameters were improved remarkably after the reconstruction (Table 2). The mean graft flow near the distal end anastomosis and the total graft flow near the proximal anastomosis prior to the side-to-side anastomosis reconstruction was 5.0 ± 2.63 ml/min and 46.21 ± 11.10 ml/min, respectively; while after the reconstruction, the value was increased to 20.71 ± 8.6 ml/min and 67.93 ± 16.68 ml/min, respectively (p < 0.0001, Table 2). The mean pulsatility index (PI) near the distal end and the proximal anastomosis was 11.31 ± 3.30 and 3.78 ± 0.93 prior to the reconstruction, respectively; while after the reconstruction, the PI value was reduced to 2.19 ± 1.01 and 2.98 ± 0.62, respectively (P < 0.001, Table 2). The graft flow parameters of the additional 16 patients who had distal end side-to-side anastomoses performed directly were also satisfactory (16.94 ± 5.58 ml/min and 53.25 ± 14.90 ml/min for distal end and total graft flow, respectively. 2.91 ± 1.11 and 3.18 ± 0.95 for distal end and total graft PI, respectively). The average time for completing a distal end side-to-side anastomosis was 3.4 ± 1.1 min. The average duration of the operation for all the patients was 4.2 ± 1.3 h. The average CK-MB level in patients reduced from 8.35 ± 4.82 ng/ml on arrival to the surgical ICU to 1.63 ± 1.01 ng/ml 48 hours after the operation, suggesting that all the patients recovered well and did not have serious perioperative complications. The patients were discharged from our hospital 7–10 days after the operation. None of the patients experienced low cardiac output syndrome, malignant arrhythmias, perioperative myocardial infarction, or other adverse complications.

**Table 2:** Comparison of the graft flow characteristics between end-to-side (ETS) and side-to-side (STS) anastomoses at the distal end of the GSV

**Patient ID**	**Graft flow**		**PI**	
	**Distal end anastomosis (ml/min) ETS/STS**	**Aortic anastomosis (ml/min) ETS/STS**	**Distal end anastomosis ETS/STS**	**Aortic anastomosis ETS/STS**
1	2/10	36/48	18.2/2.2	5.4/3.1
2	7/35	64/97	7.1/0.9	4.7/2.4
3	3/13	47/59	12.6/3.7	5.1/3.7
4	5/26	58/86	13.4/2.8	3.8/3.1
5	8/22	39/68	9.3/1.7	2.7/2.1
6	2/12	40/60	15.6/0.9	3.5/2.8
7	5/10	57/72	8.3/1.3	4.0/3.2
8	5/27	55/87	11.8/2.4	2.4/1.9
9	9/30	60/89	7.9/1.7	3.9/3.1
10	4/25	49/68	10.2/3.0	4.7/4.3
11	5/18	30/48	15.2/4.2	2.8/2.9
12	2/12	43/49	8.5/2.7	3.6/3.4
13	3/32	32/70	9.7/2.0	3.4/3.1
14	10/18	37/50	10.6/1.2	2.9/2.6
Mean ± SD	5.00 ± 2.63/20.71 ± 8.60*	46.21 ± 11.10/67.93 ± 16.68*	11.31 ± 3.30/2.19 ± 1.01*	3.78 ± 0.93/2.98 ± 0.62#

*P < 0.0001 STS vs. ETS. #P =0.0102 STS vs. ETS.

All the patients came back to follow-up visit three months after the operation. None of the patients experienced any new episode of angina since the operation. Electrocardiography during the follow-up showed significant improvement of myocardial revascularization. Echocardiography during the follow-up revealed that the mean left ventricular ejection fraction (LVEF) was significantly higher than that prior to the operation (55.23 ± 4.99% vs. 51.00 ± 1.19%, P < 0.0001), and the mean left ventricular end-diastolic diameter (LVEDD) was significantly reduced compared with that prior to the operation (52.30 ± 5.69 mm vs. 54.90 ± 5.99 mm, P < 0.05), suggesting that patient heart function was improved significantly.

## Discussion

Side-to-side anastomoses have been described to connect the left internal mammary artery (LIMA) to target coronary vessels for the purpose of reducing surgical damage on LIMA [12]. Song et al. also introduced a technique of side-to-side anastomosis with just ten proportional stitches by hand for artery grafts [13]. In vein sequential grafting, side-to-side anastomoses are usually performed for the middle anastomoses [6]. In this study, to address the issue that oversize graft-host diameter ratio at end-to-side anastomoses might induce adverse hemodynamic condition and reduce graft patency; we applied a side-to-side anastomosis technique to connect the distal end of the GSV to small target arteries in sequential CABG. We retrospectively compared the intraoperative graft flow parameters of patients who had distal end side-to-side anastomotic reconstruction and found that the reconstruction improved graft flow remarkably as compared to the end-to-side anastomoses. The comparison in this study was performed on data collected from the same patients. End-to-side anastomoses were initially performed on the 14 patients. Due to poor graft flow at the distal end, we revised the problematic distal end anastomoses into side-to-side anastomoses, resulting in significant improvement of intraoperative graft flow. We believe that the reason for the initial poor graft flow are not technical errors, instead are associated with unfavorable hemodynamic characteristics at the end-to-side anastomotic site.

Anastomotic geometry has been shown to have substantial effects on the hemodynamics near anastomotic areas. It is believed that small areas of low wall shear stress (WSS) and very few areas of high WSS gradient in the graft indicate a beneficial hemodynamic pattern for optimal graft flow [7-9]. In an end-to-side anastomosis, the vortex flow at the anastomotic heel produces large area of low WSS and high WSS gradient in the graft [9]. This type of hemodynamic pattern impedes graft flow. Multiple reports have suggested that the hemodynamic pattern associated with side-to-side anastomoses might be more favorable for graft patency compared to that of end-to-side anastomoses. Frauenfelder et al. used computational fluid dynamics to simulate pulsatile blood flow in venous grafts based on patient angiography datasets and found that flow stagnation zone exists in end-to-side anastomoses but is absent in side-to-side anastomoses [10]. Sankaranarayanan et al. analyzed the hemodynamic patterns of different anastomotic configuration by computational fluidic dynamics and found that side-to-side anastomoses have smoother blood flow and smaller spatial gradients of WSS than end-to-side anastomosis [11]. Thus, the improved graft flow dynamics we observed likely results from a smoother blood flow pattern at the reconstructed side-to-side anastomoses as compared to a vortex flow pattern at the initial end-to-side anastomoses.

In our study, the diameter ratio between the GSV and the small target coronary arteries was approximately 4:1 in the 30 patients. Graft-host diameter ratio can influence graft hemodynamics at end-to-side anastomoses substantially [2-4]. In a computational simulation model of CABG, Qiao et al. reported that large graft-host diameter ratio (1.46) produces better hemodynamics than equal or small (0.8) ratio when the diameter of a target vessel is assumed to be 2.5 mm [5]. We believe that the beneficial effects of large diameter ratio described in the report are based on the assumption that the target vessels have normal size, and the beneficial effects are limited within certain range of the diameter ratio. As the diameter ratio increases, other factors such as graft transfiguration might induce adverse effects on hemodynamics significantly, leading to an unfavorable flow patter for graft patency. In our study, the graft-host diameter ratio was approximately 4:1. Our data show that end-to-side anastomoses with such oversize diameter ratio resulted in unsatisfactory intraoperative graft flow parameters that required prompt revision, and the side-to-side anastomosis reconstruction markedly improved graft flow and PI.

### Study limitations

The patient 3-month follow-up examination showed improved cardiac function, suggesting that patient outcomes of this technique are satisfactory. Kieser et al. conducted a study of long-term follow-up of sequential CABG and found that the patency rate of side-to-side anastomoses is much higher than that of end-to-side anastomoses [14]. To assess the long-term patient outcomes of our distal end side-to-side anastomosis technique, we need to include a control group of patients receiving end-to-side anastomoses at the distal end of sequential grafts. However, in the present study, we had to revise the problematic distal end-to-side anastomoses, which produced unsatisfactory graft flow parameters, into the distal side-to-side anastomoses. Therefore, we were not able to include such control group in this study. The comparison based on the data collected from the same patients suggests that side-to-side anastomoses at the distal end of sequential vein grafts might produce a hemodynamic pattern favorable for long-term graft patency, indicating that distal end side-to-side anastomoses might be a superior anastomotic configuration than end-to-side anastomoses in general. We are now using animal models to compare the long-term graft patency of end-to-side versus side-to-side anastomosis at the distal end of grafts.

The CK-MB levels in our patients significantly dropped 48 hours after the operation, indicating the absence of serious complication associated with the surgical procedure. In this study, we were unable to directly compare CK-MB levels in patients with end-to-side anastomosis versus side-to-side anastomosis due to the absence of a control group with end-to-side anastomosis. However, according to our experience, the average CK-MB levels on arrival to the surgical ICU and 48 hours post-operation in patients undergoing routine CABG in our department are not significantly different from the CK-MB levels described in this report, suggesting that the distal end side-to-side anastomosis procedure do not appear to cause more postoperative complications compared to standard CABG procedure.

## Conclusion

In this study, we describe a technique of distal end side-to-side anastomosis in sequential CABG. Our results show that distal end side-to-side anastomoses to small target arteries can improve intraoperative graft flow markedly. Our results indicate that side-to-side anastomosis might be a promising strategy when it is necessary to connect the distal end of GSV to small target arteries in sequential CABG.

## Competing interests

The authors declare that they have no competing interests.

## Author’s contributions

HL and BX carried out the study and prepared the draft. CG performed the surgery. MG and FZ participated in data collection and analysis. JW and LD prepared the figure. YY conceived of the study, and participated in its design and coordination and helped to draft the manuscript. All authors read and approved the final manuscript.

## Pre-publication history

The pre-publication history for this paper can be accessed here:

http://www.biomedcentral.com/1471-2261/14/65/prepub
